# A Novel Soybean ERF Transcription Factor, *GmERF113*, Increases Resistance to *Phytophthora sojae* Infection in Soybean

**DOI:** 10.3389/fpls.2017.00299

**Published:** 2017-03-07

**Authors:** Yuanling Zhao, Xin Chang, Dongyue Qi, Lidong Dong, Guangjin Wang, Sujie Fan, Liangyu Jiang, Qun Cheng, Xi Chen, Dan Han, Pengfei Xu, Shuzhen Zhang

**Affiliations:** ^1^Key Laboratory of Soybean Biology of Chinese Education Ministry, Soybean Research Institute, Northeast Agricultural UniversityHarbin, China; ^2^Biotechnology Research Institute, Heilongjiang Academy of Agricultural SciencesHarbin, China

**Keywords:** *Glycine max*, *Phytophthora sojae*, GmERF113, AP2/ERF, activator

## Abstract

Phytophthora root and stem rot of soybean caused by the oomycete *Phytophthora sojae*, is a destructive disease worldwide. Ethylene response factors (ERFs) play important roles in regulating plant biotic and abiotic stress tolerance. In this study, a new ERF gene, *GmERF113*, was isolated from the highly resistant soybean ‘Suinong 10.’ Sequence analysis suggested that the protein encoded by *GmERF113* contained a conserved AP2/ERF domain of 58 amino acid and belonged to the B-4 subgroup of the ERF subfamily. Expression of *GmERF113* was significantly induced by *P. sojae*, ethylene, and methyl jasmonate. GmERF113 protein localized to the nucleus when transiently expressed in *Arabidopsis* protoplasts, could bind to the GCC-box, and acted as a transcription activator. In addition, a region of the full-length GmERF113, GmERF113-II, interacted with a basic helix-loop-helix transcription factor (GmbHLH) in yeast cells. Full-length GmERF113 also interacted with GmbHLH *in planta. GmERF113*-overexpressing transgenic plants in susceptible cultivar ‘Dongnong 50’ soybean exhibited increased resistance to *P. sojae* and positively regulated the expression of the pathogenesis-related genes, *PR1* and *PR10-1*. These results indicate that *GmERF113* may play a crucial role in the defense of soybean against *P. sojae* infection.

## Introduction

Phytophthora root rot, caused by the oomycete pathogen *Phytophthora sojae*, is a destructive disease of soybean worldwide (Wrather et al., 1997; Tyler, 2007) which commonly reduces soybean yields by between 10 and 40% (Bailey et al., 2003); severe infection can even result in a total yield loss (Zhang S.Z. et al., 2010). To better understand the resistance mechanisms of soybean plants under *P. sojae*-induced stress, it is essential to study the genes involved, as these will provide information useful for genetic engineering and breeding.

Manipulation of transcription factors is an important tool for improvement of plant tolerance against adverse environmental conditions. Numerous studies have demonstrated important roles for ethylene responsive factors (ERFs) in regulation of pathogenesis-related (PR) gene expression (Lorenzo et al., 2003; Pieterse et al., 2009; Rehman and Mahmood, 2015). The AP2/ERF transcription factor superfamily is divided into three groups, designated the AP2, RAV, and ERF families, based on their numbers of AP2/ERF domains and sequence similarities (Nakano et al., 2006). The AP2/ERF domain contains two conserved elements, the YRG and RAYD element (Okamuro et al., 1997). The YRG element contains conserved a WAAEIRD box amino acid (AA) sequence, which is involved in regulating the specificity of the DNA binding of these transcription factors (Okamuro et al., 1997). The central region of the RAYD element contains an amphipathic α-helix, which has a crucial role in mediation of protein–protein interactions (Okamuro et al., 1997). ERF family proteins contain a single AP2/ERF domain consisting of 58–59 AA residues, and are divided into the CBF/DREB and ERF subfamilies (Sakuma et al., 2002). CBF/DREB transcription factors contain valine (V) and glutamic acid (E) in the conserved DNA-binding domain, and are further sub-classified into A-1 to A-6 subgroups on the basis of their conserved domains (Sakuma et al., 2002). These proteins are primarily involved in responses to abiotic stress through recognition of dehydration-responsive or cold-repeat elements (DRE/CRT) containing the core motif, A/GCCGAC (Yamaguchi-Shinozaki and Shinozaki, 1994; Thomashow, 1999). ERF subfamily proteins contain an alanine (A, position 14) and an aspartic acid (D, position 19) in the conserved DNA-binding domain, and are further subdivided into subgroups B-1 to B-6 (Sakuma et al., 2002). ERFs bind to the *cis*-acting GCC-box (AGCCGCC) element, to mediate their crucial role in the response of plants to biotic stress (Ohme-Takagi and Shinshi, 1995; Hao et al., 1998).

Ethylene response factors function as transcription activators or repressors regulating both basal transcription levels of target genes and the activity of other transcription factors (Okamuro et al., 1997; Gu et al., 2000; Xu et al., 2007; Maruyama et al., 2013; Bui et al., 2015). For example, constitutive expression of the *AtERF1* gene elevates expression of pathogen-inducible plant defensin (*PDF1.2*), conferring resistance to *necrotrophic fungi* (Berrocallobo et al., 2002; Lorenzo et al., 2003); *NtERF5* and *TiERF1* enhance resistance to TMV (Fischer and Droge-Laser, 2004; Liang et al., 2008); and *AtERF5* and *BrERF11* increase resistance to bacteria and fungi, respectively (Son et al., 2012; Lai et al., 2013). In contrast, the transcription repressors *AtERF3/4* decrease disease resistance (Fujimoto et al., 2000; Yang et al., 2005), and *VpERF1* can increase susceptibility to both bacterial and fungal pathogens (Zhu et al., 2013).

Some ERF subfamily members also respond to phytohormones and abiotic stress in plants (Shinozaki et al., 2003; Pieterse et al., 2009; Sugano et al., 2013). Transcription of the *TaERF1* gene can be induced by exogenous abscisic acid (ABA), ethylene (ET), and salicylic acid (SA). Furthermore, overexpression of *TaERF1* activates stress-related genes, improving abiotic stress tolerance in transgenic plants (Xu et al., 2007). Expression of *GmERF7* is induced by treatment with methyl jasmonate (MeJA), ET, and ABA, and its overexpression enhances salt tolerance in transgenic tobacco plants (Zhai et al., 2013b). Moreover, overexpression of *AhERF019*, a peanut ERF gene, enhances tolerance to drought, heat, and salt stress in transgenic *Arabidopsis* (Wan et al., 2014), and mutation of ERF-associated amphiphilic repression (EAR) motif in *OsERF3* represses ET biosynthesis and drought tolerance in rice (Zhang et al., 2013). Thus, ERF proteins have important roles, not only in pathogen defense responses, but also in tolerance to various abiotic stress factors.

Many ERFs interact with other proteins to regulate the expression of their target genes (Cheong et al., 2003; Song et al., 2005; De Boer et al., 2011). Xu et al. (1998) reported that nitrilase-like protein (NLP), an enzyme involved in auxin biosynthesis, interacts with tobacco EREBP2/3 and tomato Pti4/5/6 in regulation of PR gene expression. The interaction between NtERF3 and NtUBC2 is likely to be critical for down-regulation of the repression activity of NtERF3 (Koyama et al., 2003). In addition, TaERF1 interacts with TaMAPK1, enhancing the activity of TaERF1 (Xu et al., 2007). In wheat, the ERF factor, W17, interacts with the HSP90 and PPR proteins, which may have significant transcriptional regulation roles (Qiu et al., 2011). The above examples demonstrate that the activities of ERF transcription factors can be modified by protein–protein interactions (Schwechheimer and Bevan, 1998; Li et al., 2011; Zhao et al., 2015).

In a previous study, we constructed a cDNA library from soybean ‘Suinong 10’ (which has high resistance to *P. sojae*) by suppression subtractive hybridization (SSH). The library was enriched for mRNAs encoding expressed sequence tags (ESTs) which increased in abundance during infection with *P. sojae*. In this study, an EST homologous to the AP2/ERF transcription factor, *GmERF113* (GenBank accession no. XM_003548806, NCBI protein no. XP_003548854), was isolated and its characteristics and expression patterns in response to different stress conditions analyzed, demonstrating that *GmERF113* has a role in the defense response of soybean to *P. sojae.*

## Materials and Methods

### Plant Material and Stress Treatments

The soybean cultivar ‘Suinong10,’ which is frequently used to study gene-for-gene resistance against the predominant race of *P. sojae* (race 1) in Heilongjiang, China (Zhang S.Z. et al., 2010), was used in this study. Plant stress treatments were performed at the first-node stage (Fehr et al., 1971). For hormone treatments, soybean seedlings were sprayed with 100 μM MeJA, 100 μM ABA, or 500 μM SA. ET treatment was performed in sealed plexiglass chambers by application of 2 ml of 40% Ethephon and 1 g of NaHCO_3_ dissolved in 200 ml H_2_O. During the early phase of stress treatment, leaves were collected at 0, 3, 6, 9, 12, and 24 h. Infection with *P. sojae* race 1 was performed using zoospores, following the methods described by Ward et al. (1979) and Morris et al. (1991), with minor modifications. The concentration of zoospores was estimated using a hemocytometer, and adjusted to approximately 1 × 10^5^ spores mL^-1^. Leaves were collected at 0, 6, 12, 24, 36, 48, and 72 h after treatment, immediately snap-frozen in liquid nitrogen, and stored at -80°C for quantitative real-time PCR (qRT-PCR) analysis.

The soybean cultivar ‘Dongnong 50,’ which is susceptible to *P. sojae* race 1, was used for gene transformation experiments and was obtained from the Key Laboratory of Soybean Biology in the Chinese Ministry of Education, Harbin.

### Isolation of the *GmERF113* Gene

Functional ESTs (*n* = 375) were isolated from an SSH cDNA library from the *P. sojae* resistant soybean cultivar, ‘Suinong 10,’ inoculated with *P. sojae* race 1, which was constructed in our laboratory (Xu et al., 2012). NCBI BLAST^1^ searches using these ESTs identified a cDNA clone highly homologous to plant ERF transcription factors. This clone was designated *GmERF113* (GenBank accession no. XM_003548806, NCBI protein no. XP_003548854), and amplified by RT-PCR from soybean ‘Suinong 10’ cDNA using the primers *GmERF113F* and *GmERF113R* (Supplementary Table 1) which were designed based on flanking sequences identified by searching the Phytozome database^2^. PCR was performed as follows: 94°C for 3 min, followed 35 cycles of 94°C for 30 s, 60°C for 30 s, and 72°C for 1 min, with a final extension at 72°C for 10 min. The PCR product was gel purified and cloned into the pMD18-T vector (TaKaRa, Dalian, China) and sequenced (GENEWIZ, Beijing, China). An analysis of the predicted structure of the protein encoded by *GmERF113* (GmERF113) was performed using Smart^3^. Sequence alignments were conducted using DNAMAN software^4^. A phylogenetic analysis of *GmERF113* and various heterologous AP2/ERF members was performed using the Neighbor-joining method in MEGA 5.1 software.

### Quantitative Real-Time PCR Analysis

Quantitative real-time PCR analysis was performed using a real-time RT-PCR kit (Takara, Japan) according to the manufacturer’s instructions, on a CFX96 Touch^TM^ Real-Time PCR Detection System (Bio-Rad, USA). Total RNA was extracted from soybean leaves using Trizol reagent (Invitrogen, Shanghai, China) and 1 μg was converted to first-strand cDNA using an M-MLV reverse transcriptase kit (Takara, Dalian, China). Amplification was performed using the primer pair *GmERF113-qF* and *GmERF113-qR* (Supplementary Table 1). For tissue distribution analysis, the transcript levels of the *GmEF1*β gene (GenBank accession no. NM_001248778) were used as an internal control (Supplementary Table 1 for primer sequences). The soybean housekeeping gene *GmActin4* (GenBank accession no. AF049106) was used as an internal control (see Supplementary Table 1 for primer sequences) for treatments with abiotic and biotic stresses. Relative expression levels were calculated using the 2^-ΔΔCt^ method. qRT-PCR analysis was performed using three biological replicates (i.e., RNA samples extracted from three independent plants) and three technical replicates of each biological replicate.

### Subcellular Localization of the *GmERF113* Protein

The coding region of *GmERF113* was cloned into the NcoI/SpeI sites of the pCAMBIA1302 vector using the primers, *GmERF113-GF* and *GmERF113-GR* (Supplementary Table 1), to produce the construct 35S:GmERF113-GFP; the empty vector, 35S:GFP, was used as a control. The transient expression of green fluorescent protein (GFP)-fused proteins in *Arabidopsis* protoplast cells was performed as described by Yoo et al. (2007). Transfected cells were observed using a confocal laser scanning microscope (Leica TCSSP2, Germany).

### Purification of Fusion Proteins and Electrophoretic Mobility Shift Assays (EMSAs)

The full-length coding region of *GmERF113* was inserted into the NdeI/SacI sites of the pET29b(+) vector (Novagen, Germany) using the primers *GmERF113-EF* and *GmERF113-ER* (Supplementary Table 1) to create pET29b(+)-GmERF113. The recombinant fusion plasmid was transformed into *Escherichia coli* strain BL21 (DE3). Over-expression of the cloned genes was induced using 0.5 mM isopropyl-β-D-thiogalactoside (IPTG) at 37°C for 4 h. For recombinant protein purification, bacterial cells were pelleted after induction, resuspended in 10 mL ice-cold 1×Binding Buffer (0.5 M NaCl, 20 mM Tris-HCl, 5 mM imidazole, pH 7.9), and sonicated on ice for 10 min (30 s pulse/min), until samples were no longer viscous. Following centrifugation at 12000 × *g* for 15 min at 4°C, supernatants were harvested and loaded onto His-bind Resin columns (EMD Millipore, USA). 1×Elution Buffer (0.5 M NaCl, 20 mM Tris-HCl, 1 M imidazole, pH 7.9) was added to elute the recombinant GmERF113 protein. EMSAs were performed as described by Liu et al. (2006).

### Yeast One-Hybrid Assay

To further analyze the ability of GmERF113 to bind to GCC-box motifs, coding regions of *GmERF113* were amplified and cloned into the EcoRI/BamHI sites of the GAL4 activation vector (pGADT7) (see Supplementary Table 1 for primer sequences) and the specific DNA fragments, GCC (ATCCATAAGAGCCGCCACTAAAATAAGACCGATCAA) and mutated GCC (mGCC) (ATCCATAAGA*TCCTCC*ACTAAAATAAGACCGATCAA) were cloned into the pHIS2 vector. Competent yeast cells (strain Y187) were prepared according to the Clontech Yeast Protocols Handbook. For yeast transformation, 50 μl of competent yeast cells were incubated with 100 ng of pHIS2 bait vector and 100 ng of pGADT7 prey vector, 50 μg of salmon sperm carrier DNA, and 0.5 ml of PEG/LiAc solution. Transformations were plated onto SD (-Trp, -Leu) media to select co-transformed cells and incubated at 28°C for 4 days. Transformed yeast cells were subsequently grown in SD (-Trp, -Leu) liquid media to an OD_600_ of 0.1. Aliquots of each transformed yeast cells (5 μl) were spotted on SD (-Trp, -Leu) and SD (-Trp, -His, -Leu) media plates supplemented with 100 mM 3-amino-1,2,4-triazole (3-AT) (Sigma–Aldrich). The plates were then incubated for 3 days at 28°C.

### Transactivation Assays

For transactivation assays, plasmids were constructed according to the method described by Chen et al. (2009). The β-glucuronidase (GUS) gene in pCAMBIA3301^5^ was replaced by *GmERF113*, placing the gene under the control of the cauliflower mosaic virus (CaMV) 35S promoter in the effector plasmid. To construct the reporter plasmid, four copies of the GCC-box motif and flanking sequence from the RD29A gene promoter were cloned upstream of the CaMV 35S promoter (-42 to +8). The CaMV 35S promoter contains a TATA box. This construct was inserted into pCXGUS-P, and fused to the GUS gene. Protoplast preparation and transfection were carried out according to the methods of Yoo et al. (2007). GUS activity was determined as described by Chen et al. (2006).

### Transcription Activation Assays

The full-length *GmERF113* and two cDNA fragments (*GmERF113-I* encoding AAs 1–105 and *GmERF113-II* encoding AAs 1–183) were amplified by PCR using the appropriate primers (*GmERF113-Y*, *GmERF113-IY*, and *GmERF113-IIY*; see Supplementary Table 1). PCR was carried out using KOD-Plus-Neo DNA polymerase (Toyobo), with an initial denaturation step at 94°C for 3 min, followed by 30 cycles at 94°C for 30 s, 60°C for 30 s, and 68°C for 1 min, with a final extension at 68°C for 8 min. Transcription activation assays were performed in the yeast strain, Y2HGold, which contains the *HIS3* and *ADE2* reporter genes under distinct GAL4-responsive promoter elements. Purified PCR products were inserted into the EcoRI/PstI sites of the pGBKT7 vector. Fusion plasmids and the pGADT7 vector were transformed into the yeast strain Y2HGold (Clontech). Yeast cells were selected by growth on SD (-Trp, -Leu) and SD (-Trp, -Leu, -His, -Ade) media. As positive controls, the pGBKT7-P53 and pGADT7-SV40 plasmids were inserted into yeast Y2HGold cells, while yeast cells containing the pGBKT7-Lam and pGADT7-SV40 plasmids served as negative controls.

### Yeast Two-Hybrid Library Assays

A cDNA library from soybean cultivar ‘Suinong 10’ inoculated with *P. sojae* zoospores was constructed using a Yeast Two Hybrid Library Construction kit (Clontech) in our laboratory (Dong et al., 2015). Screening for interacting proteins was performed following the manufacturer’s protocols (Clontech). Approximately 1 × 10^7^ transformants from the cDNA library were plated on SD selective (-Trp, -Leu, -His, -Ade) medium at 30°C. Yeast colonies reaching diameters > 2 mm after 3–5 days were cultured on SD selective (-Trp, -Leu, -His, -Ade) medium containing X-α-Gal (20 μg mL^-1^) and aureobasidin A (125 μg mL^-1^). Blue colonies were characterized by PCR and sequencing. Yeast Y2HGold cells carrying pGBKT7-P53 and pGADT7-SV40 served as positive controls, whereas co-expression of pGBKT7-lam and pGADT7-SV40 was used as a negative control.

Yeast two-hybrid (Y2H) experiments were performed as described by Wei et al. (2010). Full-length cDNAs of *GmbHLH*, pathogen-related protein-like (*GmPRP*), homeobox-leucine zipper protein HAT5-like (*GmHAT5*), and long-chain-alcohol oxidase (*GmFAO*) were amplified by PCR and cloned into pGADT7. Fusion plasmids and pGBKT7-GmERF113-II were co-transformed into yeast strain Y2Hgold (Clontech). After selection at 30°C, yeast colonies growing on SD (-Trp, -Leu) medium were transferred to SD (-Trp, -Leu, -His, -Ade) medium containing X-α-Gal (20 μg mL^-1^) and aureobasidin A (125 μg mL^-1^). Yeast cells carrying the pGBKT7-P53 and pGADT7-SV40 plasmids were used as positive controls, and yeast cells harboring the pGBKT7- Lam and pGADT7-SV40 plasmids were used as negative controls.

### Bimolecular Fluorescence Complementation (BiFC) Assays

For BiFC assays, the *GmERF113* gene was cloned into pSAT6-nEYFP-N1 and the full-length coding sequences of *GmbHLH*, *GmPRP*, *GmHAT5*, and *GmFAO* cDNA were also amplified by PCR and cloned into pSAT6-cEYFP-C1, respectively. These constructs were transiently transfected into *Arabidopsis* protoplasts using the polyethylene glycol method, as described by Yoo et al. (2007). Transfected cells were imaged using a TCS SP2 confocal spectral microscope imaging system (Leica).

### Soybean Transformation

The full-length coding region of *GmERF113* was PCR amplified with the primer pair *GmERF113-TF* and *GmERF113-TR* (Supplementary Table 1) and cloned into the BglII/BstEII sites of pCAMBIA3301, which contains the *bar* gene as a selective marker. The recombinant construct, *35S*:*GmERF113*, was introduced into *Agrobacterium tumefaciens* strain LBA4404 using the freeze-thaw method (Holsters et al., 1978). Cotyledonary nodes of soybean ‘Dongnong 50’ were used as explants for transformation, using the *Agrobacterium*-mediated method described by Paz et al. (2004). Phosphinothricin (8 mg L^-1^) was added to shoot proliferation medium as a selective reagent.

T_1_ transgenic soybean plants were identified by daubing phosphinothricin (125 mg L^-1^) on leaves and using PCR amplification with the primer pairs bar-F and bar-R to amplify regions of the *bar* reporter gene (see Supplementary Table 1 for primer sequences). T_2_ transgenic soybean plants were tested by PCR amplification and Southern blot hybridization using a DIG High Prime DNA Labeling and Detection Starter kit II (Roche, Germany).

### Expression Analysis of Putative GmERF113 Target Genes

*GmPR1* (XM_003545722) and *GmPR10-1* (NM_001251335), which have GCC-box motifs in their promoters, were identified as putative downstream targets of GmERF113. Relative transcript abundance of *GmERF113*, *GmPR1*, and *GmPR10-1* was compared between *35S:GmERF113* transgenic and wild-type soybean plants by qRT-PCR. The expression levels of the soybean *GmEF1*β gene were used as an internal control. Three biological replicates of qRT-PCR analyses were performed, using RNA samples extracted from three independent plants, with three technical replicates per plant.

### Assays of Pathogen Responses of Transgenic Soybean Plants

Fully expanded leaves of T_3_ transgenic soybean plants, derived from T_2_ plants identified by PCR and Southern blot hybridization, were tested by qPCR using the primer pair, *GmERF113-qF* and *GmERF113-qR* (Supplementary Table 1) and screened for resistance to *P. sojae* as described by Kim et al. (2014), with some modifications. Live leaves inoculated with *P. sojae* were covered with polythene bags to maintain relative humidity levels; culture conditions were 25°C, 90% ± 10% relative humidity, 16 h photoperiod, and 350 μmol m^-2^ s^-1^ light intensity. Soybean ‘Dongnong 50’ plants were used as controls. After 2 and 4 days, disease symptoms on each leaf were observed and photographed using a Canon IXUS 860IS camera.

To investigate the responses of plants overexpressing *GmERF113* to *P. sojae* infection, the cotyledons of T_4_ transgenic soybean plants at the first-node stage (V1) were inoculated with a suspension of *P. sojae* zoospores (Fehr et al., 1971) (concentration adjusted to approximately 8 × 10^5^ mL^-1^ using a hemocytometer), generated according to the procedure described by Ward et al. (1979), with some modifications. The relative biomass of *P. sojae* in infected cotyledons was assessed after 48 h based on the transcript levels of the *P. sojae TEF1* gene (GenBank accession no. EU079791) using soybean *GmEF1*β as a reference gene, determined according to the method described by Chacón et al. (2010) (see Supplementary Table 1 for *TEF1* and *GmEF1*β primer sequences). For each experiment, three biological replicates were performed, with three technical replicates each.

## Results

### Isolation and Sequence Analysis of *GmERF113*

**FIGURE 1:**
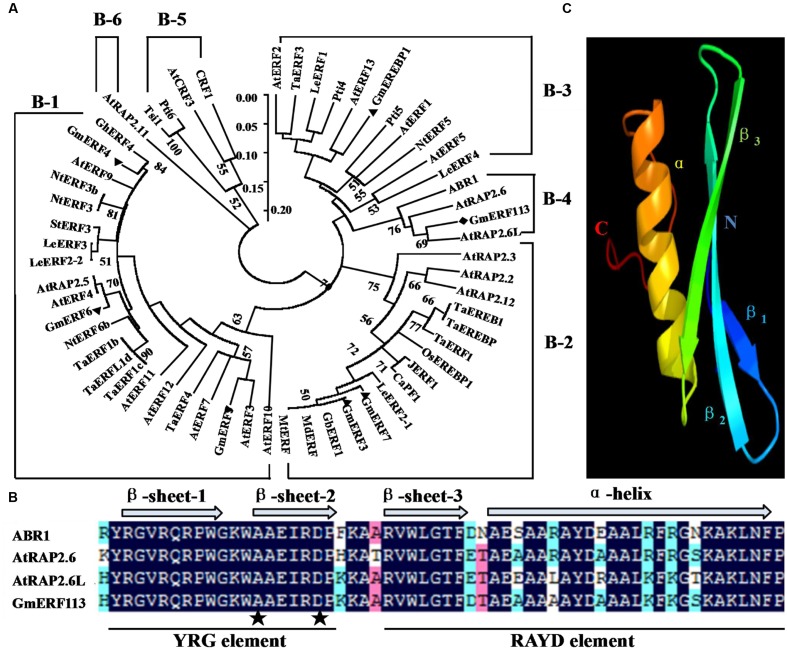
**Amino acid (AA) sequence comparison between GmERF113 and AP2/ERF-related proteins.** **(A)** Phylogenetic analysis of GmERF113 and other AP2/ERF transcription factors. The phylogenetic tree was constructed using the NJ method in MEGA5.1. The numbers on the tree branches represent values of reliability. GenBank accession numbers are as follows: AtERF1 (NP188965), AtERF2 (NP199533), AtERF3 (NP175479), AtERF4 (NP188139), AtERF5 (NP568679), AtERF7 (NP188666), AtERF9 (NP199234), AtERF10 (NP171876), AtERF11 (NP174159), AtERF12 (NP174158), AtERF13 (NP182011), AtRAP2.2 (NP566482), AtRAP2.3 (NP188299), AtRAP2.5 (EFH59179), AtRAP2.6 (NP175008), AtRAP2.6L (NP196837), AtRAP2.11 (NP197480), AtRAP2.12 (NP175794), CRF1 (NP192852), AtCRF3 (NP200141), ABR1 (NP201280), TaERF1 (AY271984), TaERF1b (DQ334413), TaERF1c (DQ334414), TaERF1d (DQ334415), TaERF3 (EF570122), TaERF4 (JX014257), TaEREB1 (AY781352), TaEREBP (AJ515477), GmERF3 (EU681278), GmERF4 (EU747723), GmERF5 (HQ896930), GmERF6 (JN416601), GmERF7 (JN416602), GmEREBP1 (AF357211), Tsi1 (AF058827), NtERF3 (AB573717), NtERF3b (AB573716), NtERF5 (AY655738), NtERF6b (AB573719), LeERF1 (AY192367), LeERF2-1 (AY192368), LeERF2-2 (AY275554), LeERF3 (AY192369), LeERF4 (NP001234313), JERF1 (AY044235), Pti4 (LEU89255), Pti5 (LEU89256), Pti6 (LEU89257), OsEREBP1 (AF193803), GhERF4 (AY781120), StERF3 (EF091875), MtERF (AES84434), MdERF (GU732435), GbERF1 (AY572463), and CaPF1 (AY246274). **(B)** Alignment of the AA sequences of the conserved AP2/ERF domain of B-4 group proteins. The three β-sheets and one α-helix of the AP2/ERF domain are marked above the corresponding sequences. The YRG and RAYD elements are indicated below the alignment. The alanine and aspartic acid residues at positions 14 and 19 in the AP2/ERF domain are marked by asterisks. **(C)** Predicted three-dimensional structure of the AP2/ERF domain of GmERF113.

The full-length cDNA sequence of *GmERF113* (GenBank accession no. XM_003548806, NCBI protein no. XP_003548854) was isolated from soybean ‘Suinong 10’ by RT-PCR. *GmERF113* maps to chromosome 16, and sequence analysis demonstrated that it was 1,259 bp in length, including 926 bp of intronic sequence. *GmERF113* had an open reading frame (ORF) of 783 bp, encoding 260 AAs with a predicted molecular mass and pI of 28.72 kDa and 6.15, respectively. The deduced GmERF113 protein contained a 58 AA conserved DNA-binding (AP2/ERF) domain, with alanine (A) and aspartic acid (D) at the 14 and 19th residues, respectively. The GmERF113N terminus included a basic AA region (R_45_KRH), predicted as putative nuclear localization signal, while the C terminus possessed a KKXX-like motif (F_256_HDK) (Supplementary Figure 1). Alignment and phylogenetic analysis indicated that *GmERF113* was most similar to previously described ERF class B-4 subgroup members (*ABR1*, *AtRAP2.6*, and *AtRAP2.6L*) (**Figure 1A**). GmERF113 shared 84.5–91.4% AA identity of the AP2/ERF domain and 30.3–45.3% overall sequence AA identity with other members of the B-4 subgroup. The AP2/ERF domain contained two conserved segments, the YRG and RAYD elements (**Figure 1B**). Based on prediction of the three-dimensional structure of GmERF113 using Phyre^6^, the protein has a long C-terminal α-helix (α) wrapped in a three-stranded anti-parallel β-sheet (β1–β3) (**Figure 1C**).

### Expression Patterns of *GmERF113* under Different Stress Conditions

**FIGURE 2:**
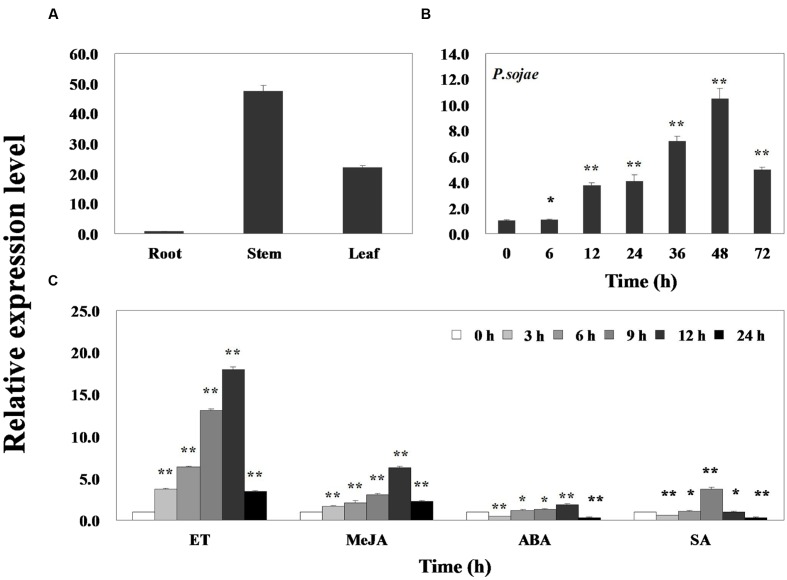
**Expression patterns of *GmERF113* in soybean.** Fourteen-day-old plants were collected for treatments and analyses. **(A)** *GmERF113* mRNA levels in different tissues of soybean. Data were normalized to expression levels of soybean *GmEF1*β. **(B)** *GmERF113* expression in soybean leaves infected with *Phytophthora sojae* for 0, 6, 12, 24, 36, 48, and 72 h. Data were normalized to expression levels of soybean *GmActin4*, and the relative expression of *GmERF113* was compared with that in mock-treated plants at the same time points. **(C)** *GmERF113* expression in soybean leaves in response to exogenous hormones: 100 μM MeJA, 100 μM ABA, and 500 μM SA and ET treatments at 0, 3, 6, 9, 12, and 24 h after the initiation of treatments. Data were normalized to expression levels of soybean *GmActin4*, and are expressed as relative to that in mock-treated plants at the same time points. Three biological replicates, with three technical replicates each were averaged and statistically analyzed using Student’s *t*-tests (^∗^*P* < 0.05, ^∗∗^*P* < 0.01). Bars indicate the standard error of the mean (SE).

Quantitative real-time PCR was performed to assess the transcript levels of *GmERF113* in soybean ‘Suinong 10’ plants. The results demonstrated that the gene was constitutively expressed, with the highest levels in the stems, followed by the leaves and roots (**Figure 2A**). *GmERF113* expression was responsive to exposure to *P. sojae*, ET, MeJA, ABA, and SA. Infection with *P. sojae* led to a gradual rise in *GmERF113* mRNA levels, with the maximum level reached after 48 h (**Figure 2B**). Treatment with both ET and MeJA led to accumulation of *GmERF113* transcripts within 3 h, with expression levels reaching a maximum level 12 h after treatment, followed by a decline. In contrast, treatment with ABA and SA induced an initial down-regulation of *GmERF113* transcription, followed by a slow increase, with maximum levels at 12 and 9 h, respectively; however, the expression of *GmERF113* was relatively low in response to these hormones, compared with that induced by exposure to *P. sojae*, ET, or MeJA (**Figure 2C**).

### Subcellular Localization of GmERF113

**FIGURE 3:**
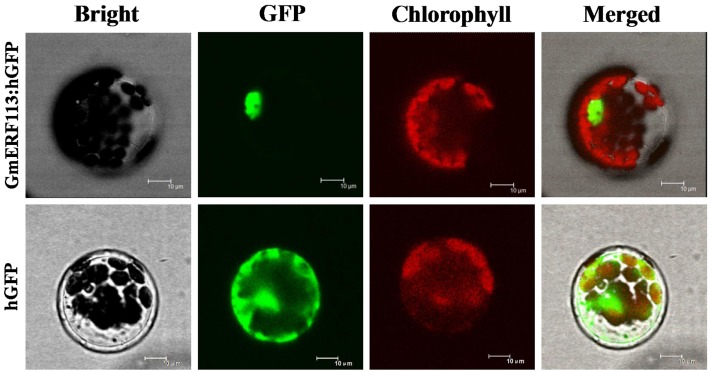
**Subcellular localization of the GmERF113 protein.** Subcellular localization of GmERF113 was determined in *Arabidopsis* protoplasts using a confocal microscope. The fluorescence distribution of control humanized (h)GFP and the GmERF113-hGFP fusion protein are shown under bright-field, GFP fluorescence (green), and chlorophyll autofluorescence (red), along with combined images. All scale bars indicate 10 μm.

To test the subcellular localization of GmERF113, a GmERF113-GFP fusion protein expressed under the control of the CaMV 35S promoter was transformed into *Arabidopsis* protoplasts. As shown in **Figure 3**, fluorescence of the control-hGFP protein was distributed throughout the cell, whereas that derived from the GmERF113-hGFP fusion protein was exclusively located in the nucleus, indicating that the GmERF113 protein exhibits nuclear localization.

### DNA Binding and Transcription Regulation Activity of GmERF113

**FIGURE 4:**
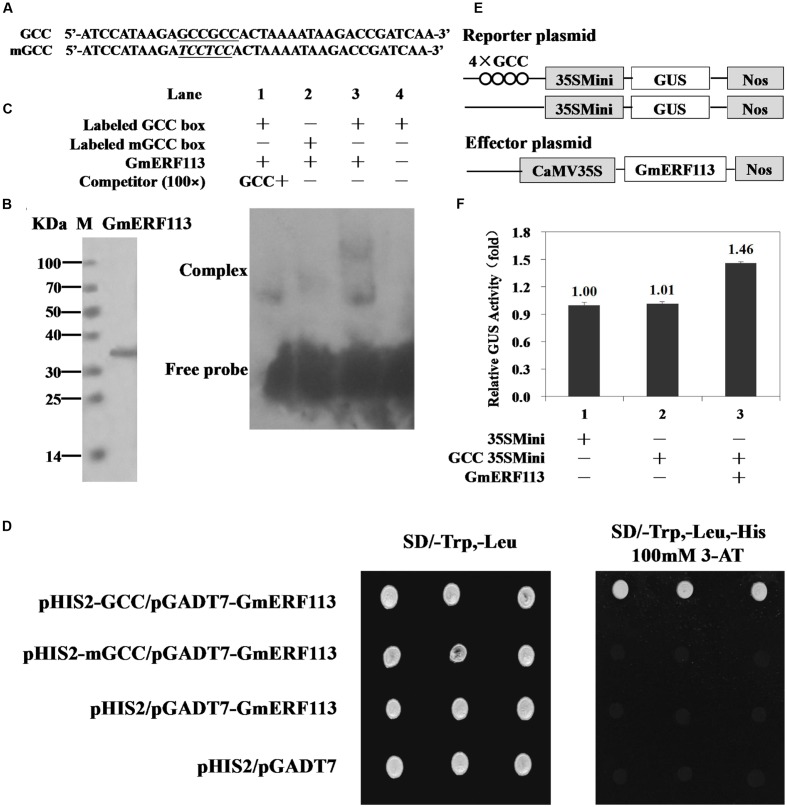
**Sequence-specific binding of GmERF113 to the GCC-box.** **(A)** Nucleotide sequences of the GCC-box and mGCC-box probes. The core GCC sequences are underlined and the mutated nucleotides in the mGCC probe are in italics. **(B)** SDS-PAGE analysis of the purified recombinant GmERF113 protein using His-Bind Kits. **(C)** EMSA showing sequence-specific binding to the GCC-box of the recombinant GmERF113 protein. Lane 1, titration with cold GCC sequence as a competitor; lane 2, labeled mGCC probe and GmERF113 protein; lane 3, labeled GCC probe and GmERF113 protein; lane 4, free GCC probe only. **(D)** The binding activity of GmERF113 to the GCC-box sequence motif in a yeast one-hybrid assay. Yeast cells were selected on SD (-Trp, -Leu) and SD (-Trp, -Leu, -His) media plates supplemented with 100 mM 3-amino-1,2,4-triazole (3-AT). **(E)** Schematic diagram of the reporter and effector plasmids. Reporter plasmids included four tandem copies of the GCC-box and 35Smini, and effector plasmids encoded GmERF113 under the control of the CaMV 35S promoter. **(F)** Relative GUS activities in transactivation assays. The numbers represent the fold increase in GUS activity compared with the control vector GCC-box/35Smini promoter (GCC 35SMini) alone. Results are presented as averages of three replicates ± standard deviation.

Electrophoretic mobility shift assays were performed to determine whether GmERF113 could bind to the *cis*-acting GCC-box DNA element *in vitro*. Nucleotide sequences used for EMSAs, GCC, and mGCC, are presented in **Figure 4A**. The recombinant GmERF113 protein was purified using His-Bind Kits (EMD Millipore, USA) (**Figure 4B**). The results of the EMSA indicated that GmERF113 could recognize and bind to the GCC-box (**Figure 4C**, lane 3), but not the mutated GCC-box (mGCC-box) (**Figure 4C**, lane 2). Competition experiments were performed to determine the specificity of the mobility shift. When the ratio of unlabeled GCC probe to labeled GCC probe was approximately 100:1, the majority of labeled GCC probe was displaced (**Figure 4C**, lane 1), indicating that the GmERF113 protein can bind specifically to the GCC-box.

To further investigate the ability of GmERF113 to bind to GCC-box elements, yeast one-hybrid assays were performed. As shown in **Figure 4D**, GmERF113 specifically bound to the GCC-box element in yeast.

To determine whether GmERF113 could act as a transcriptional activator, we performed a transactivation assay in *Arabidopsis* protoplasts using a reporter gene that had four tandem copies of the GCC-box and effector plasmids with *GmERF113* (**Figure 4E**). As shown in **Figure 4F**, GmERF113 led to a 1.46-fold higher transactivation of GCC-box-mediated transcription compared with the control, indicating that GmERF113 is able to activate transcription through this DNA element.

### Yeast Two-Hybrid Screening for GmERF113 Interacting Proteins

**FIGURE 5:**
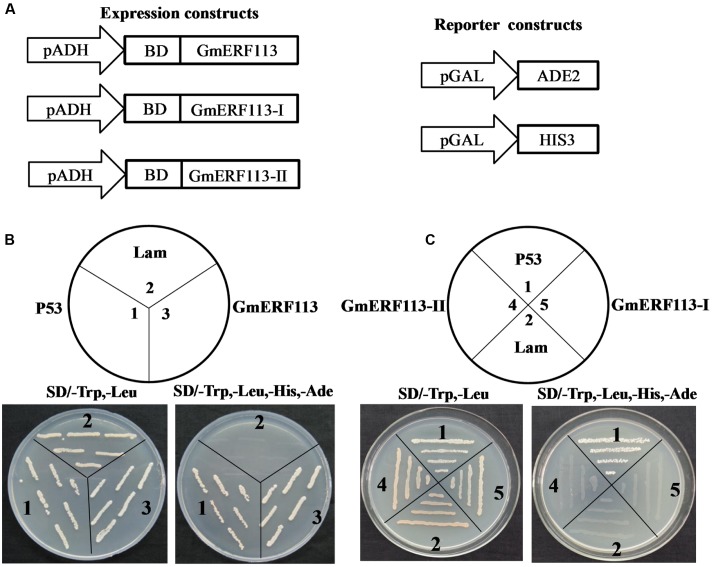
**GmERF113 transcription activation assay.** The GmERF113 transcription activation assay was performed in the Y2HGold yeast strain. **(A)** Schematic diagram of the expression and reporter constructs. The *GmERF113* gene was fused in-frame to the GAL4 DNA-binding domain (BD) expression vector and pGADT7, and transformed into yeast strain Y2HGold. The yeast strain Y2HGold contained the *ADE2* and *HIS3* reporter genes under distinct GAL4-responsive promoter elements. **(B)** The full-length GmERF113 transcription activation assay. **(C)** GmERF113-I and GmERF113-II transcription activation assay. Yeast cells were selected by growth on SD (-Trp, -Leu) and (-Trp, -Leu, -His, -Ade) media. Yeast Y2HGold cells carrying pGBKT7-P53 and pGADT7-SV40 served as positive controls, whereas co-expression of pGBKT7-lam and pGADT7-SV40 was used as a negative control (Clontech, USA).

Yeast two-hybrid analysis was performed to determine whether GmERF113 exhibited transcription activation activity in yeast cells using the expression constructs and reporter constructs (**Figure 5A**). The results demonstrated that full length GmERF113 could activate transcription in yeast (**Figure 5B**), while N-terminal fragments of the protein, GmERF113-I (AAs 1–105) and GmERF113-II (AAs 1–183) were not able to activate transcription in this context (**Figure 5C**). Therefore, GmERF113-II was used for screening the library.

Approximately 1 × 10^7^ transformants from the Yeast Two Hybrid cDNA Library were screened on SD (-Trp, -Leu, -His, -Ade) medium, and 235 selected colonies with diameters > 2 mm were further cultured on SD selective (-Trp, -Leu, -His, -Ade) medium containing X-α-Gal (20 μg mL^-1^) and aureobasidin A (125 μg mL^-1^). Among them, 53 blue colonies were characterized by analysis of their sequences using BLAST^7^. ESTs from 17 candidate genes encoding proteins that might interact with GmERF113 were listed in **Table 1**. Homology analysis demonstrated that these candidate proteins were associated with signal transduction, biotic and abiotic stress, defense response, growth regulation, and photosynthesis.

**Table 1:** Part of library screening results by yeast two-hybrid.

Gene	GenBank ID	Number of clones
Pathogen-related protein-like (*PRP*)	LOC100805630	1
Homeobox-leucine zipper protein HAT5-like (HAT5)	LOC100804450	1
Basic helix–loop–helix transcription factor (bHLH)	LOC100806368	1
Long-chain-alcohol oxidase (FAO)	LOC100779139	1
Polyubiquitin-like	LOC100791065	2
Ubiquitin-conjugating enzyme E2 7-like	LOC100782328	1
UDP-glycosyltransferase 71C3-like	LOC100792458	1
Actin -3-like	LOC100781142	1
Synaptotagmin-2-like	LOC100778906	1
Protein *S*-acyltransferase 24-like	LOC100788923	1
60S ribosomal protein L38-like	LOC100795924	1
Adenosine kinase 2-like	LOC100780391	1
Ribulose bisphosphate carboxylase/oxygenase activase, chloroplastic-like	LOC100797222	1
Chlorophyll a-b binding protein of LHCII type 1-like	LOC100796326	2
Glyceraldehyde-3-phosphate dehydrogenase chloroplastic-like transcript variant X1	LOC100806482	1
Chlorophyll binding protein 13, chloroplastic-like	LOC100779387	1
Plastid -lipid-associated protein 4, chloroplastic -like	LOC100803715	1


### Interaction of GmERF113 with GmbHLH in Yeast and *Planta*

**FIGURE 6:**
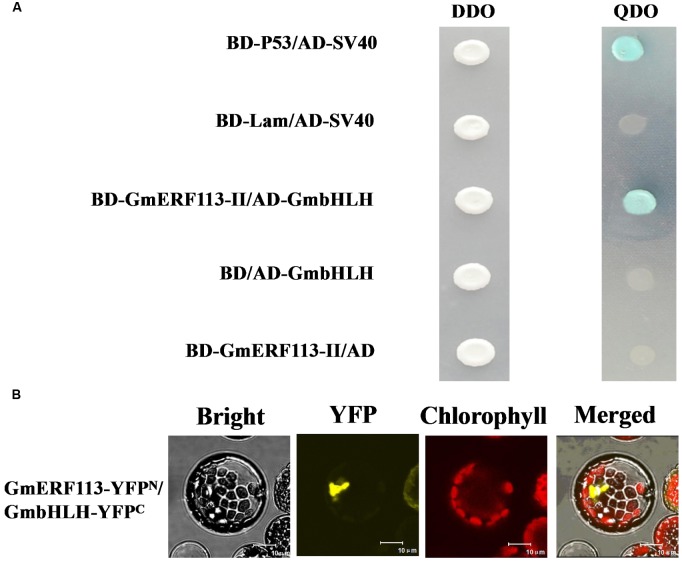
**Interaction of GmERF113 with GmbHLH in yeast cells and *planta*.** **(A)** GmERF113-II interacted with GmbHLH in yeast cells. The yeast cells were selected on SD (-Trp, -Leu) (DDO) medium and interactions were evaluated based on the ability of cells to grow on selective SD (-Trp, -Leu, -His, -Ade) (QDO) medium containing X-α-Gal (20 μg mL^-1^) and aureobasidin A (125 μgmL^-1^) for 5 days. Yeast Y2HGold cells carrying pGBKT7-P53 and pGADT7-SV40 served as positive controls, whereas co-expression of pGBKT7-lam and pGADT7-SV40 was used as a negative control (Clontech, USA). **(B)** BiFC assay of the interaction of GmERF113 with GmbHLH. *GmERF113*-YFP^N^ and *GmbHLH*-YFP^C^ were co-transfected into *Arabidopsis* protoplasts and observed using a confocal microscope. Bright-field, YFP fluorescence (yellow), chlorophyll autofluorescence (red), and combined images were visualized. Bars, 10 μm.

In order to confirm which proteins interact with GmERF113-II, four fusion genes from among the seventeen candidate genes (*GmbHLH*, *GmPRP*, *GmHAT5*, and *GmFAO*), with predicted functions related to pathogenesis and disease resistance, were selected for further investigation. Full-length cDNAs of these four genes were cloned and constructed in pGADT7. Analysis of whether these proteins had transcription activation and interaction with GmERF113 in yeast cells was performed. Our results showed that the four proteins could not activate transcription in yeast cells, and only GmbHLH interacted with GmERF113-II (**Figure 6A**), while the other three candidate proteins could not interact with GmERF113-II in yeast.

To further confirm the interaction of GmERF113 with the candidate proteins, a BiFC assay was carried out using the *Arabidopsis* protoplast transient expression system *in planta*. Obvious fluorescence was detected in the chloroplasts derived from *Arabidopsis* protoplasts cells after co-transformation of both N-terminal yellow fluorescent protein (YFP^N^)-tagged GmERF113 and C-terminal YFP (YFP^C^)-tagged GmbHLH (**Figure 6B**). However, no fluorescence was detected in *Arabidopsis* protoplast cells co-transformed with YFP^N^-GmERF113 and YFP^C^-GmPRP or YFP^N^-GmERF113 and YFP^C^-GmHAT5 or YFP^N^-GmERF113 and YFP^C^-GmFAO (data not shown). These results indicated that the GmERF113 protein and GmbHLH protein physically interacted *in plana*.

### Increased Expression of PR Genes in *GmERF113* Transgenic Soybean

**FIGURE 7:**
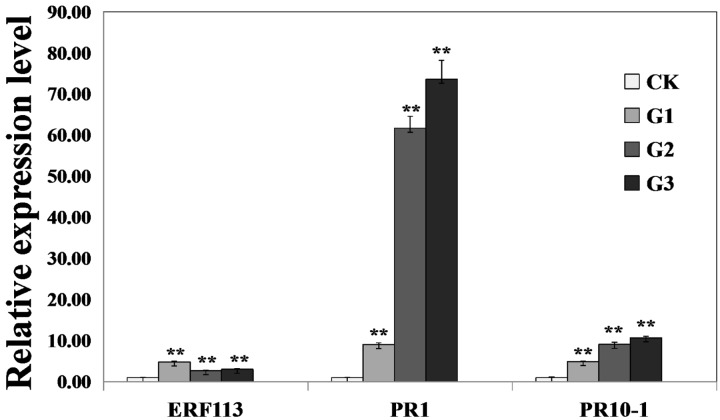
**Analysis of the expression of *GmERF113* and two PR genes in wild-type and *35S:GmERF113* transgenic soybean plants.** Relative transcript abundance of *GmERF113*, *GmPR1* (XM_003545722), and *GmPR10-1* (NM_001251335) in three transgenic lines (G1–G3) was compared with that in wild-type soybean plants. All data were normalized to levels of amplified soybean *GmEF1*β. Statistically significant differences between the *GmERF113* overexpressing transgenic lines and wild type (CK) plants were evaluated. Three technical replicates were averaged and statistically analyzed using Student’s *t*-tests (^∗∗^*P* < 0.01). Bars indicate standard error of the mean (SE).

To investigate whether GmERF113 could activate expression of downstream PR genes, the levels of *GmERF113* and two PR genes were analyzed in *35S:GmERF113* transgenic and non-transgenic soybean plants by qRT-PCR. As shown in **Figure 7**, expression levels of *GmERF113* in the three transgenic plants (G1–G3) were at least twice more than that of the control. Expression levels of *GmPR1* and *GmPR10-1*, which contained a GCC-box in their promoters, were also greatly increased in GmERF113 transgenic soybean plants, with a maximum fold-change relative to wild-type of 73 times, but barely detected in non-transgenic plants. These results indicated that the expression of *GmPR1* and *GmPR10-1* was upregulated in *GmERF113* transgenic soybean plants.

### Overexpression of *GmERF113* in Soybean Enhances Resistance to *P. sojae*

**FIGURE 8:**
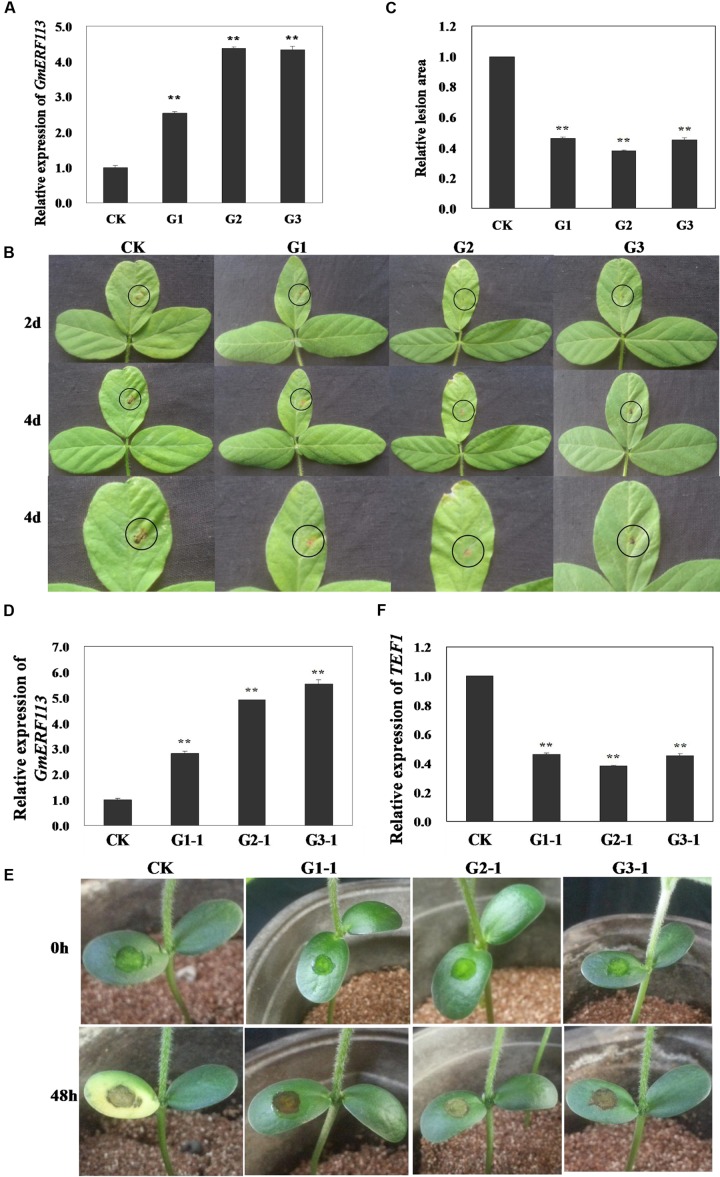
**Responses of living of *GmERF113* transgenic soybean plants to *P. sojae*.** **(A)** Quantitative real-time PCR (qRT-PCR) analysis of *GmERF113* expression levels in T_3_ transgenic soybean plants. **(B)** Disease symptoms on the leaves of T_3_ transgenic and non-transgenic lines infected with *P. sojae* race 1 inoculum at 2 and 4 days. **(C)** The lesion areas of the transgenic and non-transgenic lines were determined 4 days after inoculation with *P. sojae*. **(D)** qRT-PCR analysis of *GmERF113* expression levels in T_4_ transgenic soybean plants. **(E)** Disease symptoms on the cotyledons of T_4_ transgenic and non-transgenic lines 48 h after treatment with *P. sojae* zoospore suspension. **(F)** qRT-PCR analysis of *P. sojae* relative biomass based on the transcript level of the *P. sojae TEF1* gene in infected cotyledons 48 h after incubation with *P. sojae* zoospore suspension. The experiment was performed using three biological replicates with three technical replicates each and statistically analyzed using Student’s *t*-tests (^∗∗^*P* < 0.01). Bars indicate standard error of the mean (SE).

To determine whether overexpression of *GmERF113* can improve resistance to *P. sojae* in transgenic soybean plants, T_1_ and T_2_ transgenic plants, screened by PCR amplification and Southern hybridization, were used to generate T_3_ plants, constituting three independent *GmERF113*-overexpressing transgenic lines. The overexpression of *GmERF113* in the T_3_ transgenic soybean lines (G1, G2, and G3) was detected by qRT-PCR to investigate the response of the plants to *P. sojae* (**Figure 8A**). Four days after inoculation with *P. sojae*, the leaves of non-transgenic soybean plants showed clear, large lesions compared with those of transgenic soybean plants (**Figure 8B**); the lesion areas in transgenic soybean lines were significantly smaller than those in non-transgenic plants (*P* < 0.01) (**Figure 8C**).

Furthermore, T_4_ transgenic soybean plants (G1-1, G2-1, and G3-1) were identified by qRT-PCR (**Figure 8D**) and the relative biomass of *P. sojae* in infected live cotyledons after 48 h incubation with *P. sojae* zoospore suspensions was tested. As shown in **Figure 8E**, after 48 h of incubation with *P. sojae*, the cotyledons of transgenic soybean plants exhibited smaller lesions compared with those of non-transgenic plants. The biomass of *P. sojae*, based on the transcript levels of the *P. sojae TEF1* gene, was significantly lower in transgenic *GmERF113*-overexpressing plants than that in non-transgenics (*P* < 0.01) (**Figure 8F**). These findings demonstrated that overexpression of *GmERF113* in soybean plants increased their resistance to *P. sojae*.

## Discussion

In this study, *GmERF113*, a new member of the ERF subfamily identified in soybean, was demonstrated to increase soybean resistance to *P. sojae*. ERF transcription factors have been identified in numerous plant species, including *Arabidopsis thaliana* (Liu et al., 1998; Nakano et al., 2006; Son et al., 2012), rice (Cao et al., 2006; Zhang et al., 2013), wheat (Xu et al., 2007), cotton (Huang et al., 2007; Jin and Liu, 2008), tomato (Sharma et al., 2010), cucumber (Hu and Liu, 2011), tobacco (Fischer and Droge-Laser, 2004), Chinese wild grapevine (Zhu et al., 2013), and peanut (Wan et al., 2014), among others. To our knowledge, only six members of the ERF subfamily have been functionally characterized in soybean (Mazarei et al., 2002; Zhang et al., 2008, 2009; Zhang G.Y. et al., 2010; Zhai et al., 2013a,b; Dong et al., 2015). Among these, GmEREBP1 is assigned to the B-3 subgroup (Mazarei et al., 2002), while GmERF3 and GmERF7 (Zhang et al., 2009; Zhai et al., 2013b) are in the B-2 subgroup. These ERF proteins act as transcriptional activators and increase salt stress tolerance in tobacco. GmERF4, GmERF5, and GmERF6 are assigned to the B-1 subgroup, and contain EAR motifs, which are characteristic of ERF repressors, and can repress negative regulators of plant defense responses; thereby, constitutive expression of GmERF4 in transgenic tobacco plants increases tolerance to salt and drought stress (Zhang G.Y. et al., 2010), overexpression of GmERF6 in transgenic *Arabidopsis* enhances resistance to drought stress (Zhai et al., 2013a), and overexpression of *GmERF5* in tobacco and soybean plants improves resistance to *P. nicotianae* and *P. sojae*, respectively (Dong et al., 2015). There are three *Arabidopsis* ERFs in the B-4 subgroup, *ABR1*, *AtRAP2.6L*, and *AtRAP2.6*, which have been reported to respond to various biotic and abiotic stresses (Asahina et al., 2011; Choi and Hwang, 2011; Ali et al., 2013). As the only known soybean ERF transcription factor in the B-4 subgroup, *GmERF113* may positively regulate the expression of PR genes and enhance resistance to *P. sojae* in soybean.

Similar to other ERF proteins, GmERF113 has alanine and aspartic acid at positions 14 and 19 of the 58-AA AP2/ERF domain, suggesting that it is a member of the ERF subfamily. Sequence analysis revealed that GmERF113 has a basic AA region (R_45_KRH) toward its N-terminus which may function as a nuclear localization signal and our results demonstrate that the protein does indeed localize to the nucleus.

Using EMSAs, we verified the ability of GmERF113 to bind to GCC-box; however, the band-shift signal obtained was very weak, possibly due the *in vitro* experimental conditions being sub-optimal for protein/DNA interaction. Two specific shifted bands were observed in EMSAs, and large amounts of additional non-radioactively labeled probe was required to effectively compete with the labeled probe. The results of yeast one-hybrid assays, demonstrating that the GmERF113 protein bound specifically to the GCC-box, were more persuasive. Furthermore, a transcription activation assay showed that GmERF113 could activate GCC-box-mediated transcription. These findings suggest that GmERF113 may act as a transcriptional activator through interaction with GCC-box motifs.

Ethylene response factor subfamily genes have crucial roles in the responses of plants to biotic stress (Okamuro et al., 1997; Singh et al., 2002; Cao et al., 2006; Barah et al., 2013): overexpression of soybean *GmERF3* in transgenic tobacco led to increased resistance to *Ralstonia solanacearum*, *Alternaria alternata*, and TMV (Zhang et al., 2009); of *AtERF6* in *Arabidopsis* enhanced resistance to the fungal pathogen, *Botrytis cinerea* (Moffat et al., 2012); and of *RAP2.6* enhanced resistance against the beet cyst nematode, *Heterodera schachtii*, in *Arabidopsis* roots (Ali et al., 2013). In the present study, we demonstrated that overexpression of *GmERF113* increased soybean resistance to *P. sojae*.

Ethylene response factors regulate the expression of ET-inducible PR genes containing GCC-box sequences in their promoter regions (Ohme-Takagi and Shinshi, 1995). For example, GmERF3 can bind to the GCC-box, and overexpression of *GmERF3* in transgenic tobacco activates the expression of several PR genes, including *PR1*, *PR2*, and *PR4* (Zhang et al., 2009). Our results demonstrating significantly increased transcript levels of *GmPR1* and *GmPR10-1* in *GmERF113* transgenic soybean plants (**Figure 7**) are consistent with these published data. Our previous research confirmed that transgenic soybean plants over-expressing *GmPR10* had increased resistance to *P. sojae* (Jiang et al., 2015). We speculated that *GmPR1* and *GmPR10-1* could be direct or indirect targets of *GmERF113* and the results presented here prove that GmERF113 can positively regulate the expression of PR genes, thus improving soybean resistance to *P. sojae*.

Some *ERFs* are involved in the regulation of gene expression through interactions with other proteins or transcription factors (Gu et al., 2000; Xu et al., 2011). For example, the AP2/ERF factor, NtORC1, interacts with NtbHLH and commonly regulates the expression of genes containing G-box and GCC motifs in their promoter regions (De Boer et al., 2011). A transcriptional repressor recently identified in banana fruit, MaERF10, interacts with MaJAZ3 proteins to enforce the repression of jasmonate (JA) biosynthesis-related genes involved in MeJA-mediated cold tolerance (Qi et al., 2016). In the present study, GmbHLH was found to interact with GmERF113 using yeast two-hybrid and BiFC assays. Some bHLH proteins participate in regulation of PR gene expression (Abe et al., 1997; Friedrichsen et al., 2002); for example, the bHLH transcription factor, AtHBI1, mediates pathogen-associated molecular pattern-triggered immunity in *A. thaliana* (Fan et al., 2014). We therefore speculate that GmERF113 and GmbHLH proteins may cooperatively regulate resistance to *P. sojae* infection. Our previous research demonstrated that GmERF5, acting as a GCC-mediated transcriptional repressor, also interacted with GmbHLH and that overexpression of *GmERF5* could improve soybean resistance to *P. sojae* (Dong et al., 2015). Thus, we hypothesize that GmbHLH may play a crucial role in modulating EFR transcription factors in defense against *P. sojae* infection.

The phytohormones ET, JA, SA, and ABA are important for the regulation of defense responses in plants (Zhou et al., 1997; Pieterse et al., 2009; Seo et al., 2011; Zhu et al., 2013). Plant stress-tolerance is regulated through a network of signal transduction pathways, some of which may converge on ERF proteins through complex interactions (Zhang et al., 2004; Rehman and Mahmood, 2015). For example, AtERF4 is thought to be a key factor in the regulation of ET/ABA-dependent defense pathways and could modulate the transcription of many ET/ABA-dependent defense genes (Yang et al., 2005); GmERF3 may connect the ET, JA, and SA signaling pathways, which mediate biotic and abiotic stress responses (Zhang et al., 2009); Dong (2001) determined that two well-defined signaling pathways involved in pathogen-defense responses make use of the plant hormones SA or ET/JA, respectively; Kamsvågmagnusson et al. (2014) also reported that the expression of genes encoding ERFs is regulated in both an ET-dependent and -independent manner; while jasmonates (JAs) also play central signaling roles, using MeJA as an elicitor, in a wide range of plant resistance responses (Thagun et al., 2016). Our present study demonstrated that levels of *GmERF113* mRNA transcripts were significantly enhanced by *P. sojae*, ET, and MeJA stress; however, the observed changes elicited in response to SA and ABA stress were relatively minor. We deduce that GmERF113 may depend primarily on ET and MeJA signaling pathways, which mediate soybean responses to *P. sojae* infection.

**FIGURE 9:**
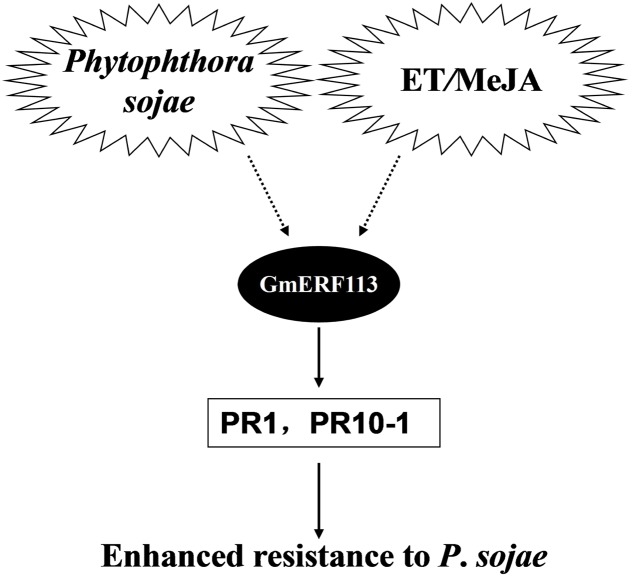
**Model of the potential role of GmERF113 as a positive regulator of soybean responses to *P. sojae* infection**.

Based on our data, we propose a model to explain the potential role of GmERF113 as a positive regulator of soybean responses to *P. sojae* infection (**Figure 9**). Expression of the *GmERF113* gene is activated by *P. sojae* infection, while exogenous ET and MeJA also induce moderate accumulation of *GmERF113* mRNA. GmERF113 is involved in the integration of signals to activate the expression of PR genes through binding to GCC-box motifs, and thereby enhances soybean resistance to *P. sojae*. Meanwhile, GmERF113 and GmbHLH may interact to cooperatively regulate the *P. sojae* resistance response. As members of the same subfamily, GmERF5 and GmERF113 have similar functions, as described above. Furthermore, expression of GmERF5 is significantly induced by ABA and SA, suggesting that this protein may be involved in ABA-mediated salt and drought tolerance. GmERF5 is the soybean EAR motif-containing ERF transcription repressor demonstrated as involved in the response to pathogen infection (Dong et al., 2015), while GmERF113 is the soybean ERF transcription activator with a crucial role in the defense of soybean against *P. sojae* infection. This study provides new insights into the mechanism by which the GmERF113 protein regulates biotic stress responses in soybean.

## Author Contributions

Conceived and designed the experiments: PX and SZ. Performed the experiments and drafted the manuscript: YZ, XCha, LJ, LD, and QC. Analyzed the data: SF, GW, XChe, DH, and DQ. Contributed reagents/materials/analysis tools: SZ and PX.

## Conflict of Interest Statement

The authors declare that the research was conducted in the absence of any commercial or financial relationships that could be construed as a potential conflict of interest.

## Footnotes

1 http://blast.ncbi.nlm.nih.gov/Blast.cgi

2 http://www.phytozome.net/

3 http://smart.embl-heidelberg.de/

4 http://www.lynnon.com/

5 http://www.cambia.org

6 http://www.sbg.bio.ic.ac.uk/phyre/

7 http://blast.ncbi.nlm.nih.gov/Blast.cgi
