# Supplementary material for: A Novel Soybean ERF Transcription Factor, GmERF113, Increases Resistance to Phytophthora sojae Infection in Soybean
Source: Front Plant Sci. 2017 Mar 7;8:299. doi: 10.3389/fpls.2017.00299 (PMC5339286; doi:10.3389/fpls.2017.00299)
Supplement: Supplementary file 1 [file Table_1.DOC]

**Supplementary Table 1.** Oligonucleotide primers used in this study.

| **Gene cloning** | *GmERF113F* | CCCATGGTAAGAGCCAAGGGGGAGAAG |
| --- | --- | --- |
|  | *GmERF113R* | GGCTAGCGGTAGGTTTTTAGCAGCACACAC |
| **qRT-PCR** | *GmERF113-qF* | GATAGCACCCTTTCTTCACCAA |
|  | *GmERF113-qR* | ATGTCTTTTTCTCCCATTTCCT |
|  | *GmPR1F* | GGCCAATACGGGGAGAATCT |
|  | *GmPR1R* | TCCAAACAACCTGAGTGTAATGC |
|  | *GmPR10-1F* | TTATTCATCCCCTGTTGCTCC |
|  | *GmPR10-1R* | ACCTCATCAATCCTGTGTTTCA |
|  | *GmActin 4-F* | GATCTACCATGTTCCCAAGT |
|  | *GmActin 4-R* | ATAGAGCCACCAATCCAGAC |
|  | *GmEF1βF* | CCACTGCTGAAGAAGATGATGATG |
|  | *GmEF1βR* | AAGGACAGAAGACTTGCCACTC |
|  | *TEF1-F* | TGATCGTGCTGAACCACCC |
|  | *TEF1-R* | CGAGCGACGGTCCATCTT |
| **GFP** | *GmERF113-GF* | CCCATGG TTATGCATCCAATCAATACTGG |
|  | *GmERF113-GR* | GACTAGT ATTCTTGTCATGAAAATCACTCC |
| **EMSA** | *GmERF113-EF* | CCATATGAAGAGCCAAGGGGGAGAAG |
|  | *GmERF113-ER* | GGAGCTCGAGCATAATGTGCGATTCTTGTC |
| **Yeast one-hybrid** | *GmERF113-Y1HF* | CCGGAATTCATGCATCCAATCAATACTGGT |
|  | *GmERF113-Y1HR* | CGGGATCCATTCTTGTCATGAAAATCACTCC |
| **Yeast two-hybrid** | *GmERF113-YF* | CATGGAGGCCGAATTCAAGAGCCAAGGGGGAGAAG |
|  | *GmERF113-YR* | GATGCGGCCGCTGCAGGGTAGGTTTTTAGCAGCACACAC |
|  | *GmERF113-IYF* | CATGGAGGCCGAATTC AAGAGCCAAGGGGGAGAAG |
|  | *GmERF113-IYR* | GATGCGGCCGCTGCAG TGGAGGAGGAGGAAGAAGAAC |
|  | *GmERF113-IIYF* | CATGGAGGCCGAATTC AAGAGCCAAGGGGGAGAAG |
|  | *GmERF113-IIYR* | GATGCGGCCGCTGCAGCCAAAGAAGCTGCGCGTAC |
|  | *GmbHLH-YF* | CATCGATACGGGATCCCTCCTTCTCTCTGTGCTTCC |
|  | *GmbHLH-YR* | GGTGCAGCTCGAGCTCCGTTATTGTTGCTTCTCGTG |
| **BiFC** | *GmERF113-BF* | CCCATGGCCTTTATGCATCCAATCAAT |
|  | *GmERF113-BR* | GGAATTCATTCTTGTCATGAAAATCACTCC |
|  | *GmbHLH-BF* | GACTCAGATCTCGAGCTCAATGGAGTCAGATCTCGAGCAG |
|  | *GmbHLH-BR* | TCTGCAGTCGACGGTACCATTGTTGCTTCTCGTGTGAACA |
| **Transformation** | *GmERF113-TF* | CAGATCTTAAGAGCCAAGGGGGAGAAG |
|  | *GmERF113-TR* | GGGTAACCGGTAGGTTTTTAGCAGCACACAC |
|  | *Bar-F* | ATATCCGAGCGCCTCGTGCAT |
|  | *Bar-R* | GGTCTGCACCATCGTCAACCACT |

**Supplementary Figure 1.** The open reading frame and deduced polypeptide sequences of GmERF113. The conserved AP2/ERF domain is shaded. The nuclear localization signal is boxed. Alanine (A) and aspartic acid (D) residues at the 14th and 19th positions, respectively of the AP2/ERF domain are single underlined and the KKXX-like motif (F_256_HDK) is double underlined.
