# Supplementary figures and images for: A Novel Soybean ERF Transcription Factor, GmERF113, Increases Resistance to Phytophthora sojae Infection in Soybean
Source: Front Plant Sci. 2017 Mar 7;8:299. doi: 10.3389/fpls.2017.00299 (PMC5339286; doi:10.3389/fpls.2017.00299)

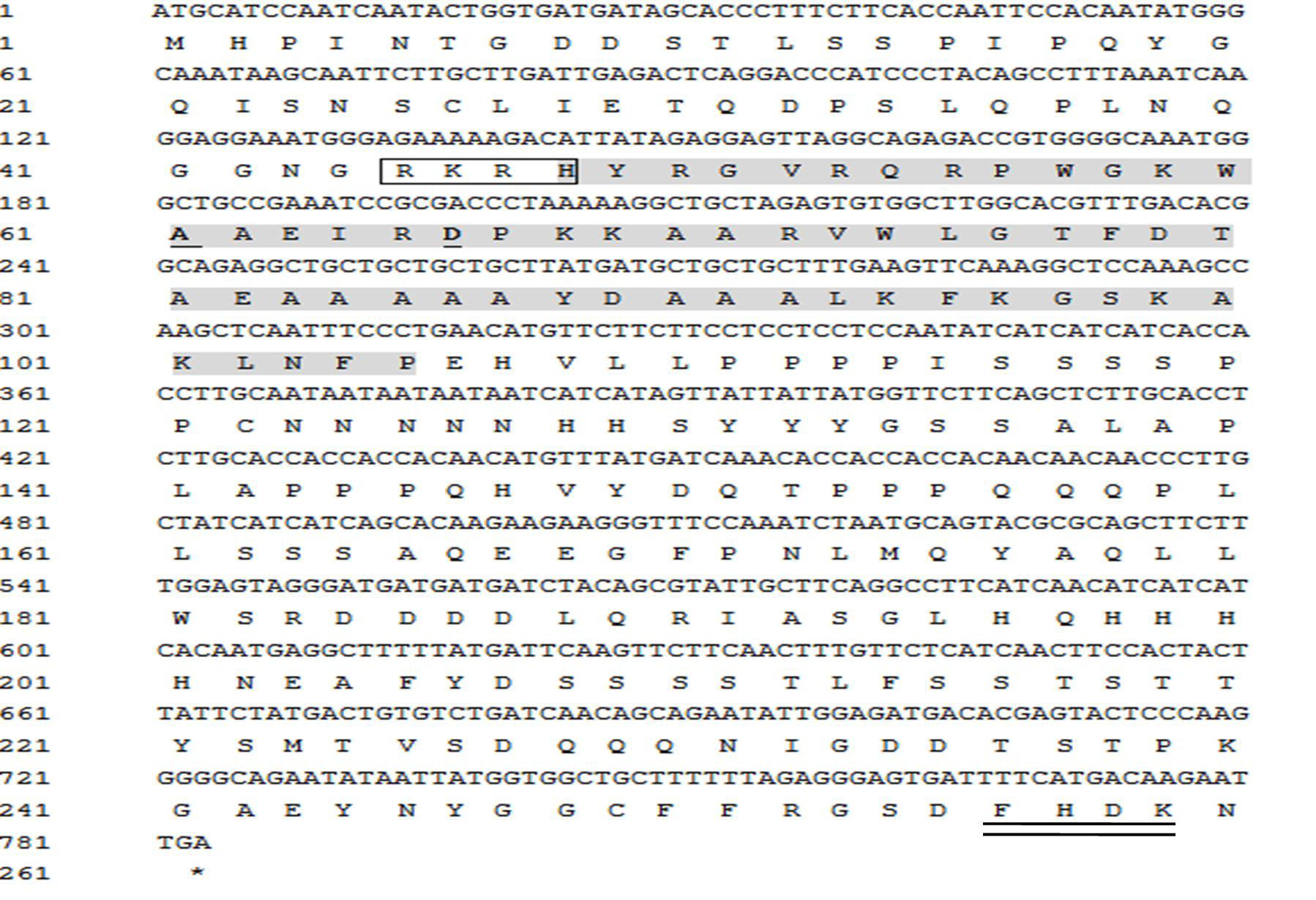

Supplement: Supplementary file 2 [file Image_1.JPEG]
